# Attitudes and Preferences of Cat and Dog Owners Towards Pet Food Quality Attributes in Türkiye

**DOI:** 10.3390/vetsci12090907

**Published:** 2025-09-18

**Authors:** Onur Erzurum, Tamer Kayar

**Affiliations:** 1 Department of Veterinary, Karapınar Aydoganlar Vocational School, Selcuk University, Konya 42400, Türkiye; 2 Department of Animal Science, Faculty of Veterinary Medicine, Aksaray University, Aksaray 68100, Türkiye; tamerkayar@aksaray.edu.tr

**Keywords:** cat, dog, feeding, food, palatability, quality

## Abstract

In recent years, pet owners have been making significant efforts to ensure the well-being of their animals. One straightforward approach to enhance their welfare involves greater attention to nutrition. Consequently, pet owners have become increasingly sensitive regarding the food they provide for their pets. The aim of this study is to examine this sensitivity by investigating the extent to which cat and dog owners prioritize quality attributes and ingredients when selecting pet food. For this purpose, a survey was conducted with pet owners (*n* = 519) who visited veterinary clinics, using a random sampling method. Of the participants, 51.25% were male and 48.75% were female, with the highest participation observed in the 18–34 age group (60.50%). Among the respondents, 64.93% owned cats, while 35.07% owned dogs. Additionally, 66.67% of pet owners reported using both wet and dry food. When selecting pet food, 42.58% of participants stated that they relied on recommendations from veterinarians. “Pet preference (palatability)” clearly emerged as the most important selection criterion, receiving the highest average score (3.90) and highlighting its decisive influence on purchasing decisions as owners appeared to prioritize what their pets liked most. In contrast, “visual appeal of the food” received the lowest score (2.47). A significant difference (*p* < 0.001) was found regarding whose recommendation influenced the purchase decision. Moreover, a negative correlation was identified between price sensitivity and other selection criteria, suggesting that price was not a primary driver in most participants’ decisions and became less important as quality-related factors gained priority. Overall, pet preference (palatability) stood out as the dominant driver of purchasing decisions.

## Simple Summary

This study examines the factors that influence cat and dog owners in Türkiye when choosing pet food. A survey of 519 pet owners visiting veterinary clinics was conducted to identify what they value most during purchase. The results showed that palatability was the top priority, followed by high protein content and the use of fresh meat. Price was considered the least important factor. Many respondents stated that they rely on their veterinarian’s advice. Physical signs of health, such as a shiny coat or good digestion, also affected food choices. These insights may help veterinarians, pet food companies, and pet care professionals develop better products and support animal health through education.

## 1. Introduction

Pet ownership has been steadily increasing worldwide, accompanied by rapid growth in the pet food industry. In Türkiye, approximately 17% of the population owned a cat or dog in 2019, rising to around 20% by 2023 [1,2]. This increase was likely influenced by the COVID-19 pandemic, which began in 2020.

Alongside the increase in pet ownership, the pet food industry has also expanded. While pet food production in Türkiye was below 90,000 tons in 2019, it reached 225,000 tons by 2022. In many developed countries, cats and dogs are regarded not merely as pets but as life companions. This anthropomorphization has led pet owners to seek more knowledge about pet care, ultimately fostering a desire to improve their companions’ welfare. One of the key ways to achieve this is by ensuring proper nutrition, which has significantly contributed to growth of the pet food industry [3,4]. In the United States, pet food expenditures have reached $65.8 billion, whereas in Türkiye, this figure stands at $127 million [5]. Across Europe, there are approximately 150 companies producing pet food, and this sector directly or indirectly provides employment for around 1,068,000 people [2]. The number of jobs generated by the pet food industry in Europe was reported to be 780,000 in 2014 and 1,000,000 in 2016 [6]. The employment rate in this sector continues to rise in parallel with the industry’s growth and the increasing number of pet owners.

Various types of pet food are available for different nutritional needs [7]. These products are offered on the market under different brand names, with varying ingredients and price ranges. Pet foods for cats and dogs are generally classified into dry food (such as extruded kibble) and wet food (such as canned food or gravy-based meals) [8,9]. In terms of consumption, dry dog food ranks first, followed by dry cat food, wet cat food, and dog treats [3]. Foods with a moisture content below 14% are classified as dry food. There are differences in the composition of dry and wet foods, mainly based on the total caloric content derived from fats, proteins, and carbohydrates. However, no definitive evidence has been provided regarding the effects of energy content or production methods on pet health [8].

Compared to the past, pet owners are now more conscious about pet food and often opt for diets that meet their pets’ specific nutritional needs. These preferences significantly influence pet food selection, and pet owners play a crucial role in decision-making. Factors such as personal research, nutritional value, perceptions of the pet food industry, and pet dietary management all contribute to these choices. Changing consumer tendencies can be challenging, but in cases where dietary modifications are necessary (e.g., organ failure), persuading pet owners becomes essential. To make these persuasion efforts effective, it is crucial to understand how pet owners make their pet food choices [10]. This is particularly important because pet food preferences directly affect pet welfare [11]. Research aimed at understanding these preferences will help pet owners make more informed and objective decisions regarding pet food [12].

Several studies have investigated the factors influencing pet owners’ food choices. For example, Selçuk and Muruz (2017) [13] highlighted the critical role of veterinarians in pet food selection and noted that pet owners pay close attention to food ingredients. Delime et al. (2020) [14] highlighted the role of the food’s scent as a key factor. Koppel et al. (2018) [15] emphasized the importance of packaging appearance. Qin (2015) [9] reported that price discounts significantly influence purchasing decisions. In contrast, Pirsich et al. (2017) [16] indicated that consumers are willing to pay higher prices for products offered by companies that prioritize animal welfare. These studies suggest that pet owners consider multiple criteria when choosing pet food, and there is no single dominant factor driving their decisions.

Previous studies on pet food purchasing preferences have been conducted in Italy [4] and the United States [12]. However, no research of similar scope and content has been carried out in Türkiye. Therefore, the present study aims to identify the main factors influencing pet food choice among cat and dog owners in Türkiye and to address this gap in the existing literature.

## 2. Materials and Methods

The data for this study were obtained from cat and dog owners in Türkiye. For this purpose, a simple random sampling method was chosen, and a face-to-face survey was conducted with 519 volunteers between February and March 2025. Participants were randomly selected from among pet owners who visited veterinary clinics in various cities in Türkiye, and the surveys were administered face-to-face. The survey was conducted with reference to a study by Vinassa et al. (2020) [4]. Prior to participation, respondents were informed about the study’s purpose, the voluntary nature of participation, and the anonymity of their responses, and informed consent was obtained.

Türkiye is geographically divided into seven regions, and participants were grouped accordingly (Central Anatolia Region, Aegean Region, Mediterranean Region, Black Sea Region, Marmara Region, Southeastern Anatolia Region, and Eastern Anatolia Region). The participants were not evenly distributed across regions.

According to FEDIAF (2024), [17] the number of individuals who own cats and dogs in Türkiye is 6,146,000. In this study, the survey was administered to pet owners who visited veterinary clinics and volunteered to participate. Since participation was based on voluntary responses from accessible individuals rather than a randomized selection from the full population, the convenience sampling method was applied. Based on a 95% confidence level and a 5% margin of error, the minimum required sample size was calculated as 385 participants. In total, 519 participants completed the survey, corresponding to an achieved margin of error of approximately 4.3%.

The collected data were analyzed using the SPSS statistical package (v21). A correlation analysis was conducted to examine the relationships between the variables measured on the Likert scale (e.g., 1 = not important and 5 = very important). Additionally, a one-way ANOVA test was performed to assess whether there were statistically significant differences among the categorical groups in the optional questions. Prior to conducting the ANOVA, assumptions of normality (Shapiro–Wilk test) and homogeneity of variances (Levene’s test) were tested and met.

## 3. Result

The responses given by the pet owners participating in the survey were calculated as percentages in order to determine the socio-demographic structure of the participants. Of the participants, 51.25% were male, 60.50% were between the ages of 18 and 34, 38.73% resided in the Central Anatolia Region, 57.42% had an associate’s degree/bachelor’s degree, 38.54% were students, and 64.93% were cat owners. The fact that most participants held an associate’s or bachelor’s degree may enhance awareness of pet nutrition (Table 1).

A majority of participants (66.67%) reported using both wet and dry food for their pets. Additionally, 31.60% indicated purchasing food from more than one source, and 42.58% reported making purchases based on a veterinarian’s recommendation (Table 2).

**Figure 1:**
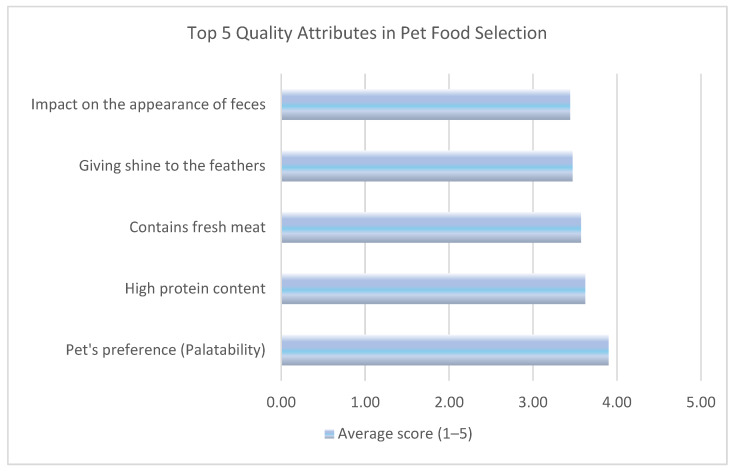
Top 5 quality attributes of selected pet food based on respondents’ average relevance scores.

Palatability emerged as the most important factor for pet owners when selecting pet food. Owners were asked to rate various features on a 1–5 Likert scale, where one represented “Not at all important” and five denoted “Indispensable.” Results showed that 30.64% of owners prioritized taste, while 21% considered price the least important factor. Based on the average scores presented in Table 3, the top three attributes in pet food selection were palatability (3.90), high protein content (3.62), and fresh meat content (3.57). Other highly rated features included giving shine to feathers (3.47) and impact on the appearance of feces (3.44). These top five quality attributes are also illustrated in Figure 1 for a clearer visual comparison.

As shown in Table 4, moderate positive correlations were found between food containing natural ingredients and both the presence of company information clearly stated on the packaging (r = 0.447) and fresh meat content (r = 0.326). Fresh meat content was also moderately correlated with meat being the main nutrient (r = 0.320) and the brightness of the pet’s coat (r = 0.308). The food’s visual appeal showed a moderate correlation with its scent (r = 0.341), while grain-free content was moderately associated with recyclable packaging (r = 0.351). Price showed weak negative correlations with natural ingredients (r = −0.190) and fresh meat content (r = −0.113).

Analysis of the purchasing habits of pet owners revealed no statistically significant differences based on geographical location, gender, age, occupation, educational attainment, the type of pet owned, the type of food procured, or the procurement channel (*p* > 0.005). However, a statistically significant difference was observed in terms of the source of recommendation for the food (*p* < 0.005).

When the results regarding who recommended the food were evaluated, statistically significant differences were observed between the veterinarian and all groups (*p* < 0.001), while no difference was observed between all other pairwise comparisons (*p* > 0.005) (Table 5).

## 4. Discussion

This study was conducted to determine the factors affecting the food preferences of cat and dog owners in Turkey. The findings indicated that pet owners consider various characteristics when selecting a food product. Consistent with the international findings of Schleicher et al. (2019), [12] who reported that animal owners primarily rely on information from veterinary health professionals (43.6%) and online resources (24.6%) when choosing food for their pets, a study by Selçuk and Muruz (2017) [13] also found that veterinarians have a significant influence on food choice (42.58%), highlighting veterinarian advice as a key determinant. This alignment suggests that veterinary recommendations play a central role in pet food purchasing decisions both in Turkey and internationally.

The analysis revealed that neither age nor educational level had a statistically significant effect on pet food purchasing behavior. This contrasts with some international studies that have reported such influences [18,19]. The lack of significance in this sample may be due to the relatively homogenous demographic profile of participants or other unmeasured factors. Future research could further investigate these variables in more diverse populations.

The preference for dry food observed in this study may reflect pet owners’ increasing awareness of oral health benefits associated with such diets. This awareness is likely influenced both by educational efforts from veterinarians and targeted marketing campaigns by pet food manufacturers emphasizing dental care advantages. Previous research has shown that owners who are better informed about oral health are more inclined to choose dry food to help prevent dental issues in their pets [20,21]. Therefore, owner knowledge and effective marketing appear to play key roles in shaping purchasing behavior towards dry pet food.

We found that the predominant locations for acquiring food were pet shops, with clinics ranking second. Oguz (2016) [22] found that cat owners’ preferred place to buy food was clinics (54.05%). In the same study, a rate of 50% was found for dogs. Prata (2022) [23] stated that pet owners in Portugal mostly buy food from supermarkets (40.3%) and then from pet shops (25%).

Palatability was identified as the most significant criterion in food preference, accounting for 30.64% of the total. Conversely, the minimal significance attributed to price (21%) is noteworthy. In contrast, Unal (2022) [24] concluded that price was of considerable importance. The observed differences in price can be attributed to variations in the income levels of the participants. In the study, the edibility of the food, which was evaluated as palatability, was found to be 53.57% for cats and 52.42% for dogs in another study [22]. Schleicher et al. (2019) [12] observed that palatability received a score of approximately 3.75, which was similar to the result in this study (3.90). The prominence of palatability may be linked to pet owners perceiving their animals’ willingness to consume food as an immediate and visible indicator of satisfaction and well-being. This perception is often reinforced by marketing strategies emphasizing taste and by cultural feeding practices where pets are considered family members. Such immediate feedback can outweigh less tangible factors like environmental sustainability or long-term health claims in owners’ decision-making processes. Similar associations between coat brightness and stool quality have been observed in other research, such as Vinassa et al. (2020) [4] which reported a relationship between coat brightness and the importance given to fecal appearance.

Correlation analyses demonstrated that characteristics such as the content of natural ingredients in the food and the utilization of fresh meat were associated with the transparent information provided by the food manufacturer. This finding suggests that consumers are demonstrating more deliberate choice behavior. The association found between fresh meat content and coat brightness, as well as the main ingredient being meat and coat brightness, may reflect pet owners’ focus on their animals’ appearance and overall well-being. Such traits are often perceived as indicators of health and vitality, underscoring the importance owners place on these factors. Similarly, Vinassa et al. (2020) [4] identified a relationship between coat brightness and the importance given to fecal appearance, reinforcing the notion that pet owners value external signs of animal health when selecting food products. The correlations observed in the present study may be explained by both nutritional and perceptual mechanisms. High-quality meat ingredients provide essential amino acids, fatty acids, and bioavailable trace minerals that help maintain skin integrity, support keratin synthesis, and enhance coat shine. As highlighted in Mota-Rojas et al. (2021) [25] deficiencies in key nutrients such as linoleic and linolenic acids can compromise skin and fur health, whereas adequate intake helps preserve cell membrane structure, water impermeability, and regulate inflammatory processes. Owners may also interpret a shiny coat and healthy stool as linked and visible indicators of well-being, which can strongly influence purchasing preferences.

The findings of the study demonstrated that environmental factors such as recyclable packaging (2.61) and grain-free food (2.62) (with recyclable packaging being one of the attributes within the broader category of environmental factors) received low scores, indicating that pet owners did not attribute sufficient importance to these features. We found that packaging received a relatively low score, consistent with the score of 1.94 reported by Schleicher et al. (2019) [12]. This situation reveals the need to raise awareness to support more environmentally friendly practices in the future [16].

This study revealed the factors influencing cat and dog owners’ pet food preferences in Türkiye, showing that palatability was the most important quality attribute, visual appeal the least important, and veterinary advice significantly influenced purchasing decisions. Additionally, most of the parameters considered important by owners were related to animal welfare indicators, including palatability, coat brightness, and normal stool appearance.

Growing awareness among consumers regarding the importance of nutritious and high-quality pet food has had a direct impact on their pet nutrition practices. It has been hypothesized that pet owners’ perception of the importance of price as low may be indicative of their tendency to prefer quality food. However, the low importance attributed to environmental factors (including recyclable packaging) signifies the necessity for enhanced awareness-raising initiatives to disseminate environmentally sustainable practices within the sector. Future studies should, therefore, aim to develop more comprehensive strategies by examining the social, economic, and environmental factors that affect food preferences in more detail. The findings of such studies will be of use to both food producers and veterinarians.

Moreover, pet food hygiene is integral to overall pet health and to the shared well-being of pets and owners. Studies show that canned dog foods are generally microbiologically safe at opening but exhibit bacterial growth in the majority of products after 24 h at room temperature, underscoring the importance of handling and storage practices [26]. In household settings, adherence to FDA–style hygiene protocols (e.g., hot-water washing and routine bowl cleaning) significantly reduces bacterial loads on food bowls, although owner awareness and long-term compliance are low [27]. Consistent with a One Health perspective, poor feeding and bowl hygiene can facilitate microbial contamination of domestic environments; contamination levels tend to be higher with wet foods and certain bowl materials, and may be mitigated by appropriate cleaning methods [28]. Emphasizing practical hygiene guidance could, therefore, reduce zoonotic risk and support the shared life quality of pets and their owners.

The majority of the participants are young people. When this situation is evaluated in conjunction with the responses given, it appears that young people are attempting to provide better care for their pets and are influenced by prevailing trends. The questions that received the highest scores in the results included those pertaining to the use of fresh meat in the production of food, the enhancement of fur shine, and the inclusion of high protein.

While this study provides valuable insights, it is important to avoid overstating the findings and to interpret the results within the context of its limitations. This study has several limitations that should be considered when interpreting the findings. First, all participants were recruited from veterinary clinics primarily located in urban areas, which may introduce sample bias. Owners who visit clinics might be more health-conscious and attentive to their pets’ welfare and nutrition compared to the general population, limiting the generalizability of the results to all pet owners in Türkiye. Second, the study relied on self-reported data, which can be subject to recall bias or social desirability bias, potentially affecting the accuracy of responses. Third, cultural factors specific to Türkiye may influence purchasing behaviors and preferences, making it difficult to generalize these findings to other countries or cultural contexts. Finally, rather than broad statements such as “consumers prefer quality over price,” it is more accurate to state that “in this sample, price was not the primary purchasing factor.” This phrasing better reflects the specific context of the study without overgeneralizing.

## 5. Conclusions

This study provides important insights into factors influencing the food preferences of cat and dog owners in Türkiye. The findings highlight that pet owners prioritize attributes such as palatability, natural ingredients, and fresh meat content when selecting pet food. Veterinary advice was also found to significantly influence purchasing decisions in this sample. However, environmental considerations such as recyclable packaging and grain-free options received comparatively low importance, indicating a need for increased consumer awareness in these areas.

The insights gained from this study have practical implications for veterinarians, pet food manufacturers, and educational campaigns aiming to promote informed and responsible pet nutrition choices. Enhanced awareness efforts focusing on environmental sustainability and oral health benefits may encourage more conscientious purchasing behaviors in the future.

Despite the valuable contributions of this research, certain limitations—such as sample bias due to clinic-based recruitment, reliance on self-reported data, and cultural specificity—should be considered when interpreting the results. Future studies are encouraged to explore these factors in more diverse populations and contexts.

## Figures and Tables

**Table 1:** Socio-demographic characteristics of survey participants (*n* = 519).

	(%)	(*n*)
Gender	(*n* = 519)	
Female	48.75	266
Male	51.25	253
Age	(*n* = 519)	
18–34	60.50	314
35–50	20.62	107
51–64	13.49	70
>64	5.40	28
Region	(*n* = 519)	
Central Anatolia	38.73	201
Aegean	14.84	77
Mediterranean	15.22	79
Black Sea	6.55	34
Marmara	13.29	69
Southeastern Anatolia	6.17	32
Eastern Anatolia	5.20	27
Level of education	(*n* = 519)	
Primary school/Middle school	10.79	56
High school/Professional qualification	26.97	140
Associate’s degree/Bachelor’s degree	57.42	298
Master	4.82	25
Occupation	(*n* = 519)	
Student	38.54	200
Housewife	10.02	52
Retired	8.29	43
Private sector	25.24	131
Public sector	7.90	41
Other	10.02	52
Animal owned (dogs and cats)	(*n* = 519)	
Cat	64.93	337
Dog	35.07	182

**Table 2:** Purchasing habits and recommendation sources of survey respondents (*n* = 519).

	(%)	(*n*)
Type of pet food purchased	(*n* = 519)	
Wet	4.43	23
Dry	28.90	150
Wet and dry	66.67	346
Preferred marketing channel for the pet food	(*n* = 519)	
Market	12.33	64
Pet shop	26.97	140
Online	13.10	68
Clinic	15.99	83
More than one	31.60	164
On whose recommendation is it purchased?	(*n* = 519)	
Friend/Relative	14.45	75
Online	9.83	51
Veterinarian	42.58	221
More than one	33.14	172

**Table 3:** Average importance scores of selected quality attributes in pet food as rated by survey participants (Likert scale 1–5).

Characteristics	1 (%)	2 (%)	3 (%)	4 (%)	5 (%)	Average Score (1–5)
Contains natural ingredients	2.12	12.72	45.09	29.48	10.60	3.34
Clear indication of manufacturer information	3.47	26.20	33.53	27.36	9.44	3.13
Pet’s preference (palatability)	1.54	4.82	26.78	36.22	30.64	3.90
Impact on the appearance of feces	5.20	15.61	29.67	29.29	20.23	3.44
Contains fresh meat	2.12	10.02	36.61	31.60	19.65	3.57
Giving shine to feathers	4.05	14.07	31.98	30.44	19.46	3.47
The main ingredient is meat	2.12	19.85	36.99	26.97	14.07	3.31
Smells good	8.29	26.78	34.68	20.04	10.21	2.97
High protein content	2.31	9.44	34.68	31.21	22.35	3.62
Visual appeal	18.69	42.77	18.30	13.29	6.94	2.47
Grain-free	10.40	37.57	35.26	12.72	4.05	2.62
Recyclable packaging	17.53	33.33	27.17	14.26	7.71	2.61
Being a well-known brand	7.90	17.34	33.72	24.86	16.18	3.24
Price	21.00	19.65	24.08	18.69	16.57	2.90

**Table 4:** Spearman correlation coefficients (r) between respondents’ survey responses (*n* = 519).

	Natural Ingredients	Company Information	Palatability	Feces Appearance	Fresh Meat	Shiny Coat	Meat Main Ingredient	Smells Good	Protein Content	Visual Appeal	Grain-Free	Recyclable	Brand
Company information	0.447 **												
Palatability	0.285	0.106											
Feces appearance	0.227	0.247	0.258										
Fresh meat	0.326 **	0.274	0.116	0.271									
Shiny coat	0.159	0.180	0.203	0.240	0.256								
Meat main ingredient	0.156	0.188	0.170	0.143	0.320 **	0.308 **							
Smells good	0.169	0.181	0.138	0.055	0.102	0.209	0.189						
Protein content	0.230	0.225	0.131	0.265	0.246	0.238	0.294	0.229					
Visual appeal	0.105	0.117	0.073	0.090	0.096	0.139	0.073	0.341 **	0.133				
Grain-free	0.056	0.175	0.002	0.092	0.180	0.062	0.190	0.167	0.195	0.226			
Recyclable	0.027	0.189	−0.025	0.099	0.152	0.159	0.064	0.215	0.138	0.236	0.351 **		
Brand	0.104	0.156	0.108	0.109	0.131	0.209	0.187	0.159	0.198	0.254	0.252	0.281	
Price	−0.190	−0.049	−0.065	−0.106	−0.113	−0.131	−0.008	0.097	−0.016	0.123	0.097	0.067	0.137

All values in the table represent correlation coefficients (r). Correlations were interpreted using standard cutoffs: r ≤ 0.1 = weak, r > 0.3 = moderate, r > 0.5 = strong. Correlations above 0.3 are highlighted in bold. ** = *p* < 0.001

**Table 5:** One-way ANOVA results for the question “Whose advice was the food purchased based on?” (*n* = 519).

	N	Mean	Std. Deviation	Minimum	Maximum	F	*p*
Relatives/Friend ^B^	75	3.049	0.411	2.14	4.00	8.829	0.000
Online ^B^	51	3.041	0.535	1.79	3.86		
Veterinarian ^A^	221	3.296	0.422	1.93	4.21		
More than one ^B^	172	3.145	0.484	2.00	4.79		

Groups labeled with different letters (A, B) indicate statistically significant differences based on post hoc test results. Groups sharing the same letter are not significantly different from each other.

## Data Availability

The original contributions presented in this study are included in the article. Further inquiries can be directed to the corresponding author.
